# The relationship between adolescent legal cognition and academic burnout: the moderating role of satisfaction with the natural environment

**DOI:** 10.3389/fpsyg.2025.1594695

**Published:** 2025-10-14

**Authors:** Yandong She, Shuhui Xu

**Affiliations:** ^1^Department of Education, Wenzhou University, Wenzhou, Zhejiang, China; ^2^Institute of Higher Education, Wenzhou University, Zhejiang, China; ^3^Department of Psychology, Wenzhou University, Wenzhou, Zhejiang, China

**Keywords:** legal cognition, academic burnout, natural environment satisfaction, legal education, interdisciplinary perspective

## Abstract

**Background:**

Academic burnout is common among adolescents and is linked to adverse academic and behavioral outcomes. Although poor academic performance is a known risk factor for juvenile delinquency and legal cognition has been identified as a protective factor against delinquent behavior, the relationship between legal cognition and academic burnout has not been examined.

**Methods:**

In June 2024, 518 in-school students (secondary and university) in Zhejiang Province, China completed measures of legal cognition, academic burnout, and natural-environment satisfaction. Data were analyzed in SPSS: Pearson correlations, hierarchical regression controlling for gender, age, and parental education, and simple-slope tests with 5,000 bootstrap resamples to probe significant interactions.

**Results:**

Legal cognition correlated negatively with academic burnout (*r* = −0.16, *p* < 0.001) and positively with natural-environment satisfaction (*r* = 0.34, *p* < 0.001); burnout correlated negatively with natural-environment satisfaction (*r* = −0.12, *p* = 0.010). In regression models, legal cognition negatively predicted burnout (β = −0.19, *p* < 0.001). Importantly, the interaction between legal cognition and natural-environment satisfaction was significant (β = −0.08, *p* = 0.038), and simple-slope tests showed that the inverse association between legal cognition and burnout was stronger at higher levels of natural-environment satisfaction.

**Conclusion:**

Legal cognition is inversely associated with adolescent academic burnout, and this protective effect is amplified when students report greater satisfaction with their natural environment. Findings support integrated interventions that combine legal-education components with environmental improvements to mitigate academic burnout.

## 1 Introduction

Academic burnout, defined as a stress reaction to prolonged academic demand that depletes psychological and cognitive resources (Wang et al., 2023; Salanova et al., 2010), is common among adolescents and university students and carries adverse educational and public-health consequences (e.g., elevated emotional exhaustion, cynicism, impaired performance, and increased dropout risk). Large reviews and recent large-scale studies document substantial prevalence and downstream harms of school/university burnout (Kaggwa et al., 2021; Liu et al., 2023; May et al., 2015; Fiorilli et al., 2017). Given these scope and impact considerations, scalable, school-feasible upstream strategies (as complements to clinical services) are especially valuable (Bresó et al., 2011; Dubuc-Charbonneau and Durand-Bush, 2015; Jing et al., 2024).

This study tests two complementary, school-implementable resources that may reduce burnout risk: legal cognition (strengthened via legal education) and satisfaction with the natural environment (improved via campus/landscape interventions). We further control for key covariates (gender, age, parental education) given their established links to developmental trajectories and academic outcomes (Eagly and Wood, 2016; Metcalfe and Mischel, 1999; Davis-Kean, 2005).

### 1.1 Legal cognition and academic burnout

Legal cognition—encompassing abstract understandings of law’s values and concrete awareness of rights and obligations—is a core product of legal socialization (Xu and Yan, 2022). Although research has emphasized legal cognition’s role in compliance and delinquency (Hirschi, 1969; McLean et al., 2019; Mazerolle et al., 2021), it also plausibly affects processes central to burnout. First, role clarity and procedural fairness: internalized beliefs about fair procedures and institutional legitimacy reduce ambiguity and perceived injustice in school contexts, lowering stress and emotional exhaustion (Reicher and Emler, 1985; Główczewski and Burdziej, 2023; Li et al., 2024). Second, self-regulation and engagement: perceiving school authority as legitimate supports adaptive coping, sustained engagement, and better self-regulation—proximal mechanisms that protect against exhaustion and disengagement (Maslach et al., 2001; Meier and Schmeck, 1985). Integrating these pathways reconceptualizes legal cognition as a psychosocial resource that can diminish academic burnout by shaping appraisal, coping, and engagement.

*Hypothesis 1*. Legal cognition negatively predicts students’ academic burnout.

### 1.2 The moderating role of natural-environment satisfaction

Satisfaction with the natural environment denotes subjective evaluations of local green space, air quality, and related ecological attributes. Theories of stress recovery and attention restoration posit that restorative environments replenish depleted cognitive and emotional resources (Ulrich et al., 1991; Kaplan and Kaplan, 1989; Hartig et al., 2014). Empirical syntheses confirm that nature exposure reduces stress and improves affect and attention—capacities that are taxed by academic demands (Brown et al., 2013; Jimenez et al., 2021; Gaekwad et al., 2023).

We propose a concise moderating framework with two complementary mechanisms. First, restorative buffer: higher environmental satisfaction restores cognitive/emotional resources, enabling students to better mobilize the self-regulatory and coping benefits conferred by legal cognition. Second, contextual amplification: a supportive physical environment increases receptivity to school socialization and thus enhances the translation of legal-cognitive resources into adaptive behavior and sustained engagement; conversely, poor environmental satisfaction may blunt these effects.

*Hypothesis 2*. Satisfaction with the natural environment moderates the association between legal cognition and academic burnout, such that the negative relation is stronger at higher levels of environmental satisfaction.

### 1.3 The current study

This study integrates two underexamined determinants—legal cognition and satisfaction with the natural environment—to explain adolescent academic burnout. Theoretically, it tests whether legal cognition functions as a psychosocial resource (via perceptions of legitimacy and procedural fairness) that reduces burnout, and whether restorative environmental resources condition that relationship. Practically, the study evaluates a tractable, school-embedded intervention logic: strengthening legal cognition through education and improving environmental satisfaction are complementary, scalable strategies to reduce academic exhaustion. We therefore test the following hypotheses:

*Hypothesis 3*. Legal cognition negatively predicts students’ academic burnout.

*Hypothesis 4*. Satisfaction with the natural environment moderates the relationship between legal cognition and academic burnout, such that higher environmental satisfaction strengthens the protective effect of legal cognition.

## 2 Method

### 2.1 Participants

Participants were 518 in-school adolescents (secondary school and university) in Zhejiang Province, China, recruited via convenience (non-probabilistic) sampling based on accessibility and willingness. The target population comprised approximately 6,000 in-school adolescents aged 12–24 in the province. Inclusion criteria were current enrollment, age 12–24, and informed consent (parental consent for minors); exclusion criteria were inability to complete the survey or missing/invalid responses. The sample included 173 males and 345 females (*M* = 18.23, SD = 3.38).

An *a priori* power analysis conducted with G*Power indicated that a sample of about 300 participants would be required to achieve 80% power at α = 0.05 to detect small-to-moderate effects (Cohen, 1992). This estimation is consistent with methodological heuristics for factor analysis, which recommend a minimum of 5–10 participants per item or at least 300 participants for stable solutions (Comrey and Lee, 1992). The achieved *N* = 518 therefore exceeds both the power analysis requirement and methodological guidelines, providing adequate power and model stability.

The 12–24 age range is treated as a single “youth” cohort because all participants are in-school students sharing core role demands and living contexts, consistent with criminological and developmental conventions defining youth as under 25 (Bu, 2022).

### 2.2 Measures

#### 2.2.1 Adolescent academic burnout scale

Adapted from Zhao Yufen’s Middle School Students’ Academic Alienation Scale (Zhao, 2019), prioritizing items overlapping with emotional exhaustion—the central dimension of burnout (Maslach et al., 2001; Meier and Schmeck, 1985). The 17-item scale (e.g., “I think learning is a burden”) uses a 5-point Likert scale; higher scores indicate greater burnout. Items were reviewed by experts for conceptual relevance. Cronbach’s α = 0.951.

#### 2.2.2 Adolescent legal cognition scale

Adapted from Xu Shuhui’s College Students’ Legal Cognition Scale (Xu, 2019), 29 items assessing concrete (e.g., rights/obligations) and abstract (e.g., legal values) cognition. 5-point Likert scale; higher scores reflect greater legal cognition. Cronbach’s α = 0.987.

#### 2.2.3 Satisfaction with natural environments questionnaire

Adapted from Yunnan Province Environmental Satisfaction Survey (Liu and Li, 2020), 14 items (e.g., air quality, green space). 5-point Likert scale; higher scores indicate greater environmental satisfaction. Cronbach’s α = 0.831.

A pre-test (*n* = 45) was conducted across the three instruments to assess item clarity and reliability, leading to minor revisions of selected items.

Covariates. Gender, age, and parental education were included due to their influence on burnout and legal cognition.

### 2.3 Procedures and data analysis

Data were collected in June 2024 via paper-and-pencil surveys administered by a graduate student in classrooms during scheduled breaks. Participation was voluntary, no incentives were offered, and parental consent was obtained for minors. Standardized instructions emphasized independent responding, anonymity, and spatial separation to reduce bias.

Analyses were conducted in SPSS 21.0. Descriptive statistics and Pearson correlations were computed. Common method bias was evaluated via procedural safeguards and Harman’s single-factor test (12 factors, first factor = 31.23% < 40%).

Moderation analyses used Hayes’ PROCESS macro (Model 1; Hayes, 2013), chosen for direct estimation of interactions with bias-corrected bootstrap CIs. Continuous predictors were grand-mean centered; missing data were minimal and handled via listwise deletion. Reported estimates include 95% bias-corrected bootstrap CIs (5,000 resamples). SEM was considered for future work to explicitly model measurement error.

## 3 Results

### 3.1 Descriptive statistics and correlation coefficients among variables

The correlation analysis revealed that academic burnout was significantly negatively correlated with both legal cognition and natural environment satisfaction. Legal cognition showed a significant positive correlation with natural environment satisfaction (see Table 1).

**Table 1:** Descriptive statistics and correlation analysis of research variables (*N* = 518).

Variables	*M ± SD*	1	2	3	4	5
1. Age	18.23 ± 3.38	1				
2. Gender	–	−0.16^*^	1			
3. Legal cognition	4.61 ± 0.67	−0.10^*^	−0.07	1		
4. Academic burnout	2.45 ± 0.88	0.14^**^	−0.03	−0.16^***^	1	
5. Natural environment satisfaction	3.48 ± 0.69	−0.14^**^	0.02	0.34^***^	−0.12^**^	1

***p < 0.001, **p < 0.01, *p < 0.05.

### 3.2 Moderating effect of natural environment satisfaction

The moderating effect was tested using the Process macro (Model 1) after standardizing all variables prior to analysis (Hayes, 2014). The results, as shown in Table 2, indicated that, after controlling for gender, grade level, and the education levels of both parents, legal cognition significantly negatively predicted academic burnout. The interaction term between natural environment satisfaction and legal cognition also significantly negatively predicted academic burnout, suggesting that natural environment satisfaction moderated the relationship between legal cognition and academic burnout.

**Table 2:** The moderating role of satisfaction with the natural environment in the relationship between legal cognition and academic burnout.

Variables	Academic burnout			
	β	*t*	*p*	CI
Constant	−0.56	−2.03	0.043	[−1.09, −0.03]
Gender	0.03	−0.41	0.680	[−0.22, 0.14]
Age	0.04	2.77	0.058	[0.01, 0.06]
Father’s education level	−0.09	−1.89	0.059	[−0.19, 0.00]
Mother’s education level	0.06	1.29	0.198	[−0.03, 0.17]
Legal cognition	−0.19	−3.71	<0.001	[−1.30, −0.09]
Natural environment satisfaction	−0.05	−1.15	0.249	[−0.149, 0.04]
Legal cognition × natural environment satisfaction	−0.08	−2.46	0.014	[−0.14, −0.01]
*R* ^2^	0.063			
*ΔR* ^2^	0.013		0.014	
*F*	4.928		<0.001	

*N* = 518. Standardized regression coefficients with 95% confidence intervals (CI) are reported. The introduction of the interaction term resulted in a significant increment in explained variance, Δ*R*^2^ = 0.013, *p* = 0.014.

**Figure 1:**
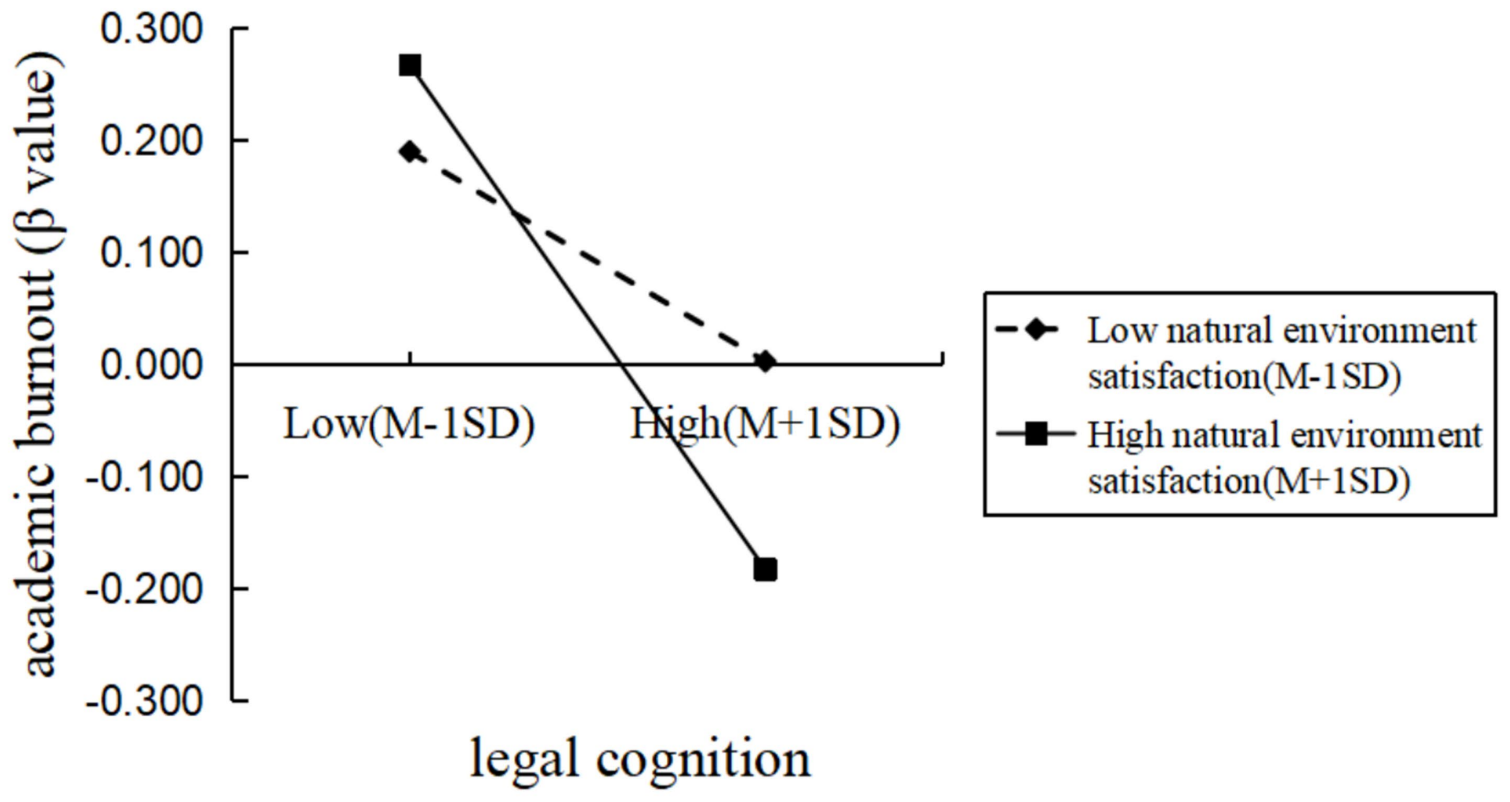
The moderating effect of satisfaction with the natural environment on the relationship between legal cognition and academic burnout.

To further explore this effect, participants were divided into high and low natural environment satisfaction groups based on one standard deviation above and below the mean. Simple slope analysis revealed that, in the low natural environment satisfaction group, legal cognition significantly negatively predicted academic burnout. In the high natural environment satisfaction group, legal cognition also significantly negatively predicted academic burnout, but the predictive effect was stronger. This suggests that as participants’ satisfaction with the natural environment increased, the negative predictive effect of legal cognition on academic burnout was further strengthened (see Figure 1).

## 4 Discussion

This study found that legal cognition negatively predicts academic burnout, but effect sizes were small (β = −0.19; interaction β = −0.08) and the model accounted for only ~6.3% of variance, so findings warrant cautious interpretation. Prior research links poor academic performance to both burnout and juvenile delinquency (Lee, 2013; Duru et al., 2014; Oyoo et al., 2020), indicating that legal cognition is likely one modest protective factor among many. Because burnout can increase vulnerability to deviant behavior, our results provide preliminary (non-causal) evidence that stronger legal-cognitive resources are associated with lower burnout, but they do not establish a definitive pathway from legal cognition to reduced delinquency via reduced burnout.

Perceptions of law and legal authority foster self-regulation by signaling procedural fairness and legitimacy, which in turn promote rule-compliant behavior (Tyler, 2006, 2009). In the school context, internalized legal-norms support students’ engagement and adaptive study behaviors, whereas academic burnout reflects depletion of these regulatory and motivational resources; thus higher legal cognition is expected to protect against burnout. Moreover, abstract legal beliefs—akin to a belief in a just world—emphasize fairness and proportionality and may buffer stress responses (Lerner and Simmons, 1966; Yu et al., 2023); teacher perceptions of legal legitimacy further shape students’ compliance and engagement (Fine and van Rooij, 2021).

The present study found that satisfaction with the natural environment moderates the relationship between legal cognition and academic burnout. Specifically, higher environmental satisfaction strengthens the negative association between legal cognition and academic burnout, consistent with our hypothesis. The natural environment is an important source of vitality, which can enhance students’ engagement and academic performance while reducing burnout and dropout risk (Wefald and Downey, 2009). Moreover, both natural and artificial environments have been shown to promote creativity relative to neutral settings (Chulvi et al., 2020). Satisfaction with the natural environment reflects individuals’ subjective evaluation of environmental quality, and, according to Ulrich’s theory of restorative attention, higher satisfaction facilitates cognitive restoration and energy recovery (Ulrich, 1983). This restorative context may support the deployment of self-regulatory and engagement processes associated with legal cognition, thereby reinforcing its protective effect against burnout. However, given the small interaction effect observed, this moderating influence should be interpreted as modest and preliminary.

Several limitations warrant caution. The cross-sectional, convenience sample limits causal inference and generalizability, and reliance on self-report measures raises shared-method bias; future studies should incorporate teacher reports, behavioral outcomes, and objective environmental indices (e.g., GIS green-space, air-quality data). The model accounted for only ~6.3% of variance, so additional predictors (e.g., personality, peer climate, teaching quality) deserve examination. We therefore recommend longitudinal or experimental designs, broader predictor sets, and multimethod measurement to replicate and clarify mechanisms. Finally, future work would benefit from measuring burnout and academic alienation concurrently to delineate overlap and unique predictors.

## 5 Conclusion and recommendations

This study provides preliminary evidence that stronger legal cognition is associated with lower academic burnout and that perceived natural-environment quality modestly strengthens this association. Although effect sizes are small, the findings point to two complementary, school-feasible strategies: (1) pilot brief, curriculum-embedded legal-education modules that emphasize procedural fairness, rights/obligations, and self-regulation; and (2) implement low-cost environmental enhancements (e.g., increased greenery, accessible restorative spaces, basic indoor air-quality measures) to boost restorative opportunities. These combined strategies should be evaluated via rigorous trials using multimethod outcomes (self-report, teacher/behavioral data) and objective environmental measures to establish effectiveness and scalability. Cross-sector collaboration among educators, administrators, and planners is encouraged to support implementation and policy alignment.

## Data Availability

The original contributions presented in the study are included in the article/supplementary material, further inquiries can be directed to the corresponding author.
